# Development of Pesto Sauce with Moringa Leaves and Baru Almonds: A Strategy to Incorporate Underutilized Ingredients with Nutritional and Sensory Viability

**DOI:** 10.3390/foods14132377

**Published:** 2025-07-04

**Authors:** Renata Moraes Brito, Eliara Acipreste Hudson, Jaqueline de Paula Rezende, Andréa Alves Simiqueli, Maria do Carmo Gouveia Peluzio, Márcia Cristina Teixeira Ribeiro Vidigal, Ana Clarissa dos Santos Pires

**Affiliations:** 1 Food Technology Department, Federal University of Viçosa, Av. P. H. Rolfs s/n, Viçosa 36570-900, MG, Brazil; renata.m.brito@ufv.br (R.M.B.);; 2 Food Science Department, Federal University of Lavras, Trevo Rotatório Professor Edmir Sá Santos s/n, Campus UFLA, Lavras 37203-202, MG, Brazil; jaquelinerezende@ufla.br; 3 Pharmacy Department, Instituto of Life Sciences, Federal University of Juiz de Fora, Campus Governador Valadares, Governador Valadares 35032-620, MG, Brazil; 4 Nutrition and Health Department, Federal University of Viçosa, Av. P. H. Rolfs s/n, Viçosa 36570-900, MG, Brazil

**Keywords:** unconventional food plant, volatile compounds, headspace solid-phase microextraction technique, rate-all-that-apply, sustainability

## Abstract

The growing demand for healthy and sensorially pleasing foods is accompanied by increasing sustainability concerns among consumers and industry. Therefore, exploring native and underutilized resources for traditional preparations is important. This study evaluated the incorporation of Moringa oleifera leaves and baru almonds (*Dipteryx alata*) in pesto sauce, comparing them to the traditional recipe regarding composition, color, total phenolics, volatiles, sensory characteristics, and acceptability. The following four formulations were developed: basil with cashew nuts (B/CN); basil with baru almonds (B/BA); and two versions with 50% basil replaced by moringa, combined with cashew (BM/CN) or baru (BM/BA). BM/BA presented the highest protein content (9.0%), compared to B/CN (7.9%). BM/CN showed a greener color. BM/CN and BM/BA showed total phenolics and antioxidant capacities similar to B/CN. BM/BA showed elevated condensed tannins (113.28 mg CE/100 g). All samples contained 1,8-Cineole and linalool, key to the aroma of basil. Pesto with moringa and/or baru showed good sensory acceptance, rated as “liked moderately”, with no difference from the conventional version (*p* > 0.05). There were no differences in the basil aroma, nutty flavor, or greasiness. Pesto sauce is a promising matrix for incorporating regional, underused ingredients such as moringa leaves and baru almonds, expanding their potential in new food development.

## 1. Introduction

The growing pursuit of a healthy lifestyle, along with modern habits, has increased the demand for ready-to-eat foods that are both nutritious and palatable [1]. To meet consumer expectations, the food industry continues to innovate, yet concerns remain regarding its environmental sustainability [2]. Therefore, there is a need to develop foods that are healthy, sustainable, and sensorially acceptable.

One promising approach is the use of underutilized plant resources with high nutritional value and low environmental impact [3]. This strategy gains importance considering the projected 17% growth in the global population between 2025 and 2050 [4], which will challenge current food production systems.

Unconventional food plants (UFPs), traditionally consumed in local diets, have emerged as important ingredients for sustainable food development [5]. Many UFPs are rich in fiber, lipids, vitamins, and phenolic compounds [6,7], making them suitable for functional food applications. *Moringa oleifera* and *Dipteryx alata* (baru almond) stand out due to their nutritional quality and adaptability to diverse environments, reinforcing their potential in sustainable formulations.

*Moringa oleifera*, known as the “tree of life,” is native to India and widely cultivated in tropical regions due to its adaptability to adverse environmental conditions [8]. Its dehydrated leaves exhibit a high protein content (24–31%, *w*/*w*), along with vitamins A, C, and E, and minerals such as iron (25.6 mg/100 g) and calcium (2185 mg/100 g) [9,10]. These nutritional attributes have sparked interest in the use of moringa as an ingredient in food formulations aimed at improving nutritional value [11]. Studies using the leaves in extract or flour form have also demonstrated antioxidant and preservative effects [12,13,14], in addition to influencing technological and sensory attributes in various foods [15,16], further supporting its potential as a versatile ingredient in food formulations.

Likewise, *Dipteryx alata*, a species native to the Brazilian Cerrado biome, produces almonds known for their high content of unsaturated lipids (approximately 80%, *w*/*w*), relevant levels of calcium, iron, and zinc [17], as well as their vitamins and phenolic compounds, among which tannins are noteworthy [6]. In addition to their nutritional value, baru almonds have demonstrated potential benefits against metabolic disorders [18,19], colorectal cancer [20], and chronic kidney disease [21] in preclinical studies. From a technological perspective, the pleasant sensory characteristics of baru almonds, particularly their flavor and texture, support their use as ingredients in food products [22,23].

Although there are studies on the individual use of *Moringa oleifera* leaves and baru almonds, the combined application of these ingredients in food formulations, particularly in ready-to-eat products, remains underexplored. Moringa has commonly been used in the form of extracts or powders, and the incorporation of whole leaves represents a promising alternative that can expand its uses in popular foods, contributing to the promotion of biodiversity, dietary diversification, and the development of sustainable products.

Pesto is a traditional Italian sauce for pasta, and its basic formulation includes basil, cheese, extra virgin olive oil, and pine nuts. This sauce enjoys global popularity, being extensively diffused [24]. Moreover, its formulation allows for various adaptations, contributing to the diversification of pesto sauce. The variability in pesto composition results in a wide range of pesto sauces available in the market, driven by the increasing interest of consumers in this food [25]. Various studies have been conducted with different ingredients for pesto preparation, including coriander (*Coriandrum sativum* L.) [24], faba bean seeds (*Vicia faba* L.) [26], kale, and broccoli [27]. However, the nutritional potential of UFPs, such as moringa and baru almond, as ingredients in pesto sauce remains unexplored.

Thus, the aim of this study was to investigate the potential use of moringa leaves and baru almonds as alternative ingredients to basil and pine nuts in the preparation of pesto sauce. The formulations developed were compared to the Brazilian traditional version of pesto, in which cashew nuts are commonly used as a substitute for pine nuts due to their wide availability in the country. To achieve this, the physicochemical properties, phytochemicals, microbiological aspects, sensory characteristics, and acceptability were evaluated.

## 2. Materials and Methods

### 2.1. Obtaining Ingredients

Moringa leaves, from the 2023 harvest, were collected in the city of Viçosa, Minas Gerais, Brazil (20°45′14″ S, 42°52′55″ W). Baru almonds were obtained from the local market in the city of Aragarças, Goiás, Brazil (15°53′50″ S, 52°13′48″ W). The other ingredients, including basil, roasted cashew nuts, Parmesan cheese, extra virgin olive oil, garlic, and salt, were purchased from the local market of Viçosa, Minas Gerais, Brazil.

### 2.2. Pesto Sauce Formulations

The experiment followed a completely randomized design with three replications. The treatments consisted of four pesto formulations, in which basil was partially replaced (50%) in order to preserve the typical sensory characteristics of the sauce [28]. In addition, cashew nuts were also replaced. The formulations were as follows: a traditional one (B/CN) with basil (22%), cashew nuts (11%), olive oil (54%), Parmesan (10%), garlic (2%), and salt (1%); one with cashew nuts fully replaced by baru almonds (B/BA); and two additional formulations with a 50% substitution of basil leaves with moringa leaves, plus cashew nuts (BM/CN) or baru almonds (BM/BA) (Table 1).

Basil and moringa leaves were sanitized in 200 mg·L^−1^ sodium hypochlorite for 30 min and rinsed. Baru almonds were roasted at 140 °C for 30 min, and the skins were manually removed before being used in the preparation of the formulations [17]. All ingredients were blended (5 min), packed in polypropylene containers, and stored at −18 °C until analysis.

### 2.3. Determination of the Centesimal Composition of the Ingredients and the Pesto Sauces

The centesimal composition of the ingredients was assessed using 10 g of cashew nuts and baru almonds, 5 g of moringa and basil leaves, and 5 g of pesto sauce samples. The analysis was performed by quantifying moisture, ash, lipids, and proteins, following the methodology established by the Association of Official Analytical Chemists [29]. Carbohydrate content was calculated by difference.

### 2.4. Color Mensurement Procedures

Color measurements were performed using a ColorQuest XE colorimeter (HunterLab, Reston, VA, USA), standardized with white and black references. Readings of 10 g of pesto sauce were performed in triplicate at 25 °C. Color was expressed as L* (luminance, ranging from black to white), a* (chromaticity, ranging from green to red), and b* (chromaticity, ranging from blue to yellow), with C* (saturation index) and h° (Hue angle) calculated from the a* and b* values. The total color difference (∆E) was determined using Equation (1), which is as follows:

(1) $$\Delta E={\left[({L_{i}^{*}{-L}_{t}^{*})}^{2}+({a_{i}^{*}{-a}_{t}^{*})}^{2}+({b_{i}^{*}{-b}_{t}^{*})}^{2}\right]}^{0.5}$$

where $L_{t}^{*}$, $a_{t}^{*}$, and $b_{t}^{*}$ are the color parameters of the traditional pesto sauce formulation and $L_{i}^{*}$, $a_{i}^{*}$, and $b_{i}^{*}$ are the parameters of other pesto sauce formulations [30].

### 2.5. Phytochemical Compounds

Total phenolic compounds and condensed tannins were extracted following Klug et al. [26], with modifications. A total of 1g of pesto was homogenized with 10 mL methanol using an ultrasonic bath (Branson 3510-DTH, Gaithersburg, MD, USA) for 10 min, then centrifuged at 15,000× *g* for 5 min. This process was repeated twice, and the supernatants were combined and adjusted to a final volume of 25 mL.

#### 2.5.1. Total Phenolic Compounds

Total phenolic compounds were quantified using the Folin–Ciocalteu method [31], with absorbance measured at 740 nm (PerkinElmer Lambda 35 UV-VIS, Shelton, CTt, USA). A gallic acid standard curve (0.01–0.08 mg⸱mL^−1^) was used.

#### 2.5.2. Condensed Tannins

Condensed tannins were determined by the vanillin–HCl method [32]. A total of 1 mL of extract was mixed with 2.5 mL of 1% vanillin and 2.5 mL of 8% HCl in methanol and incubated at 30 °C for 20 min under agitation. Absorbance was measured at 500 nm using a UV-VIS spectrophotometer (PerkinElmer Lambda 35), with methanol as a blank. Results were expressed as mg catechin equivalents per gram of sample (mg CE/g).

### 2.6. Antioxidant Capacity

The antioxidant capacity was assessed using the same extracts utilized in the determination of total phenolic compounds.

#### 2.6.1. Free Radical ABTS Capture

The ABTS assay was performed as described by Re et al. [33]. A 7 mmol/L ABTS solution with 2.45 mmol/L potassium persulfate was incubated in the dark for 16 h at 25 °C. For analysis, 30 μL of extract was added to 3 mL of ABTS solution, incubated at 30 °C for 25 min, and then absorbance was measured. Results were expressed using a Trolox standard curve.

#### 2.6.2. Ferric-Reducing Antioxidant Power (FRAP)

The FRAP assay followed the methodology described by Benzie and Strain [34]. The FRAP reagent was prepared by mixing acetate buffer (300 mmol/L, pH 3.6), TPTZ (10 mmol/L in 40 mmol/L HCl), and ferric chloride (20 mmol/L). For the assay, 2400 μL of reagent, 240 μL of distilled water, and 80 μL of extract were incubated at 37 °C for 15 min, and absorbance was read at 593 nm. Results were expressed using a Trolox standard curve.

### 2.7. Determination of Volatile Compounds

Volatile compounds in pesto sauces were analyzed using headspace solid-phase microextraction (HS-SPME) coupled with GC-MS (QP2010SE, Shimadzu, Tokyo, Japan). First, the pesto sample (5 g) in a 20 mL headspace vial with a PTFE septum was incubated at 30 °C for 120 min to reach headspace equilibrium. Then, a DVB/CAR/PDMS fiber (Supelco, Darmstadt, Germany) was exposed to headspace for 30 min for the extraction of volatile compounds. After extraction, compounds were desorbed for 3 min in a GC column (SLB-5MS, 30 m × 0.25 mm × 0.25 μm). The injection port operated in splitless mode, with split flow activated after 2 min at 250 °C. Helium was used as the carrier gas (1.2 mL/min). The MS operated in electron impact mode (70 eV), with an ion source temperature of 200 °C. The column temperature program was 40 °C (5 min), increasing to 160 °C at 5 °C/min (held for 10 min), then rising to 250 °C at 10 °C/min. Volatile compounds were identified by comparing mass spectra and retention indices (RIs) with spectral libraries (Wiley 229, FFNSC13) and the literature [20]. RIs were calculated using an alkane standard (C8–C17) according to Equation (2), which is as follows:

(2) $${RI}_{X}=\left({RI}_{Z}+100\right)\times [({RT}_{x}-{RT}_{Z})/({RT}_{z+1}-{RT}_{z})]$$

where *RI_X_* and *RT_x_* are the index and retention time of the evaluated compound, respectively. *RI_z_* and *RT_z_* refer to the index and retention time of the standard hydrocarbon whose retention time precedes that of the evaluated compound, respectively. *RT_z+1_* indicates the retention time of the standard hydrocarbon following the evaluated compound.

### 2.8. Microbiological Analyzes

The microbiological quality of the formulations was assessed using 25 g of sample by counting Enterobacteriaceae, filamentous fungi, and yeasts (CFU/g), and by detecting *Salmonella* spp., following APHA [35] guidelines.

### 2.9. Sensory Analysis

The formulations under study were evaluated in relation to acceptability, purchase intention, and sensory characteristics by 100 consumers of pesto sauce (65 women and 35 men) aged between 18 and 63 years old. All evaluations were carried out in the Sensory Analysis Laboratory, in individual cabins, and under white light. Four coded formulations were presented monadically, with 7 g of pesto sauce served on bread across four sessions. Acceptability was assessed for appearance, aroma, texture, flavor, and overall impression using a 9-point hedonic scale (1 = “disliked extremely” to 9 = “liked extremely”), while purchase intention was rated on a 5-point scale (1 = “certainly would not buy” to 5 = “certainly would buy”).

Sensory characterization was performed using the “Rate-All-That-Apply” (RATA) test [36]. Consumers were presented with four coded formulations, in a monadic and randomized order, alongside a food base and an evaluation form containing 20 descriptive terms (characteristic appearance of fresh herbs; characteristic color of basil pesto; phase separation; olive oil aroma; nut aroma; basil aroma; fresh herbs flavor; garlic flavor; light; basil flavor; salty taste; bitter taste; nut flavor; rancid; sour taste; cheese flavor; greasy; granular; uniform; dry). The sensory terms were determined based on the literature and preliminary tests [25,37]. Consumers selected applicable terms and rated intensity on a 3-point scale (0 = not applicable, 1 = low, 2 = medium, 3 = high). Term order was randomized per consumer, while sample order remained fixed. The form utilized for sensory analysis is displayed in Supplementary Materials Figure S1.

This experiment was conducted in accordance with ethics approval number 6054921.

### 2.10. Statistical Analysis

Data were analyzed using a one-way ANOVA and Tukey’s test, with a significance level of 0.05, using R software (version 4.2.2). Furthermore, correlations among obtained data were conducted using the Pearson’s correlation coefficient (r). Additionally, sensory data were analyzed using principal component analysis (PCA).

## 3. Results and Discussion

### 3.1. Centesimal Composition

The proximate composition analyses of the main pesto ingredients (moringa and basil leaves, cashew nuts, and baru almonds) was determined to obtain detailed information on the contribution of ingredients to the pesto sauce formulation (Table 2). The centesimal composition of the four pesto sauce samples is presented in Table 3.

In general, the centesimal compositions of the pesto sauces varied with the inclusion of moringa leaves and baru almonds. The moisture content of the B/BA sample (25.04%) was higher compared to other formulations (*p* < 0.05). This increase in moisture can be attributed to the combination of ingredients, basil leaves, and baru almonds, which showed higher moisture compared to moringa leaves and cashew nuts. The results indicate that the combination of moringa leaves and baru almonds was decisive in increasing the carbohydrate and protein content of the pesto sauce (BM/BA) compared to the traditional formulation (B/CN). This is due to the higher concentration of carbohydrates and proteins in moringa leaves and baru almonds in contrast to basil leaves and cashew nuts, respectively. On the other hand, due to the lower lipid content of baru almonds (38.3%) compared to cashew nuts (49.1%), the lipid content in the baru almond sauce formulations showed a reduction. Therefore, the incorporation of moringa leaves and baru almonds into pesto sauce led to changes in the macronutrient composition, contributing to a higher protein density and reduced fat content, which may support the development of products with an improved nutritional profile.

### 3.2. Color Analysis

Color analysis is important to evaluate how the alternative ingredients, such as moringa leaves and baru almonds, used in the pesto sauce formulation influence its colorimetric parameters. The inclusion of moringa leaves and baru almonds in the formulations significantly altered the chromatic properties of the pesto sauces (Table 4). All samples exhibited L* values below 50, indicating a dark appearance [38], with the BM/BA formulation showing the lowest lightness value (*p* < 0.05). This reduction may be attributed to the high chlorophyll content of moringa leaves, a pattern previously observed in other foods enriched with this ingredient, such as gluten-free bread [39]. In addition to darkening the product, this characteristic may contribute to a more intense and visually appealing color.

The hue angle (h°) values obtained for the pesto sauce samples were around 90°, indicating a hue close to yellow tending toward green. All formulations differed significantly from each other (*p* < 0.05), demonstrating that the variables under study imparted a distinct color to each pesto sauce within the visible spectrum. The hue angle variability among the formulations was notably influenced by the inclusion of moringa leaves and baru almonds. Specifically, the sample combining moringa leaves and baru almonds (BM/BA) showed a more pronounced green hue compared to the other samples, followed by the formulation in which 50% of the basil leaves were replaced by moringa leaves, with the cashew nuts preserved (BM/CN). This can be attributed not only to the higher chlorophyll content in moringa leaves but also to their lower degradation during processing compared to basil chlorophyll [40]. These observations suggest potential advantages such as improved color retention and stability, which may enhance the visual appeal and shelf life in products incorporating moringa leaves.

The chroma value (C*) was independent of pesto sauce composition (*p* > 0.05). This outcome is favorable as it indicates that, despite the substitution of traditional ingredients, the samples retained a uniform level of color intensity [41]. However, ∆E values exceeding 2 indicate distinguishable color changes between the traditional formulation (B/CN) and B/BA, BM/CN, and BM/BA that can be perceptible to the human eye. The incorporation of baru almonds, and especially moringa leaves, resulted in a significant reduction in the a* value, consequently enhancing the green hue of the pesto sauce. This color change may be interesting due to the desirable color profile for this type of food. Therefore, integrating these UFPs into pesto sauce formulations can represent a favorable strategy, exerting a positive influence on its color attributes.

### 3.3. Bioactive Compounds and Antioxidant Capacity

Phenolic compounds have been studied in products containing nuts and herbs due to their ability to act as antioxidants, and their additional potential health benefits [42,43,44]. The inclusion of different ingredients in pesto sauce can change the content and profile of phenolic compounds in the final product. Table 5 displays the content of phenolic compounds and the antioxidant capacity in the pesto sauce formulated with different ingredients.

The pesto sauce formulations B/CN, BM/CN, and BM/BA exhibited higher levels of total phenolic compounds and antioxidant capacities determined by the ABTS and FRAP methods (*p* < 0.05) compared to the B/BA formulation. Specifically, the pesto sauce incorporating moringa leaves and baru almonds (BM/BA) demonstrated an approximately 57% increase in total phenolic compounds compared to the formulation with basil and baru almonds (B/BA). Although the literature reports high TPC values in moringa (245 mg GAE/g) [45], in contrast to basil (3 a 17 mg GAE/g) [46], and relatively low TPC in baru almonds (5 mg GAE/g) [20] and cashew nuts (1 mg GAE/g) [47], our results revealed an unexpected pattern. The combination of basil leaves and baru almonds resulted in the lowest TPC concentration and antioxidant activity. This result may be attributed to an antagonistic interaction between basil and baru, probably involving specific interactions that led to the degradation or reduced extractability of phenolic compounds.

Similar results for total phenolic compounds were found in a study of pesto sauce prepared from basil grown in soil (159.31 mg/100 g) [24]. On the other hand, in the study of pesto made from faba beans seeds, higher values for total phenolic compounds (298 mg GAE/100 g) were reported [26]. It is important to emphasize that the total phenolic compounds are influenced by various factors, such as cultivar type, growing conditions, processing, and extraction methods [48].

Concerning condensed tannins, the BM/BA sample exhibited the highest value among all pesto sauces. The obtained value (113.28 mg EC/100 g) indicates that nearly 70% of total phenolic compounds comprise condensed tannins. These compounds confer antioxidant capacity and find applications across diverse industries, such as pharmaceutical, medical, and food [49]. However, despite the recognized functional benefits associated with condensed tannins, there is a challenge related to their concentration in foods given their inherent ability to precipitate proteins and induce an astringent flavor, which may pose a sensory disadvantage [50].

The antioxidant capacity refers to the ability of compounds to act as proton and electron donors to stabilize free radicals. The ABTS and FRAP methods, involving electron transfer reactions, are frequently employed to quantify this parameter [51]. Antioxidant capacity values obtained by these methods ranged from 167.67 mg TEAC/100 g (B/BA) to 304.94 mg TEAC/100 g (B/CN) for ABTS and from 75.63 mg TEAC/100 g (B/BA) to 133.25 mg TEAC/100 g (BM/BA) for FRAP. Formulations with higher phenolic compounds concentrations, i.e., B/CN, BM/CN, and BM/BA, exhibited increased antioxidant capacity determined by both ABTS and FRAP methods. The presence of highly reducing functional groups, such as phenolic groups in phenolic compounds, enables them to donate electrons or hydrogen when in contact with an oxidizing agent, resulting in the formation of oxidized products of bioactive compounds [52]. In this context, the antioxidant capacity of the ingredients used can be attributed to the presence of various phenolic compounds identified in each of them. Basil, for instance, contains phenolic acids such as caftaric, caffeic, ferulic, chicoric, and rosmarinic acids [53,54]. In moringa leaves chlorogenic and gallic acids are the main phenolic acids, along with flavonoids such as catechin, kaempferol, rutin, naringenin, luteolin, and myricetin [7]. In cashew nuts the predominant compounds are (+)-catechin and gallic acid, whereas in baru almonds p-coumaric, isoferulic, ellagic, and gallic acids have been identified, along with their derivatives including gallic acid esters and gallotannins [20,47]. Positive correlations were observed between phenolic compounds content in B/CN (0.97, *p* < 0.05) and BM/CN (0.69, *p* < 0.05) samples and FRAP values. However, the BM/BA sample showed a negative correlation (−0.75, *p* < 0.05), indicating the presence of other bioactive compounds influencing the result. Notably, BM/CN and BM/BA samples demonstrated similar concentrations of bioactive compounds and antioxidant capacity as the traditional sample (B/CN).

### 3.4. Analysis of Volatile Compounds

Pesto sauce is distinguished by its intense aroma, derived from the combination of ingredients, especially herbs, olive oil, and nuts. The aroma is a crucial factor influencing the overall acceptance of pesto. Therefore, comprehending the volatile compounds in pesto sauce and evaluating how the inclusion of diverse ingredients can impact these compounds is essential.

HS-SPME analysis of the pesto sauce formulations revealed an average identification of 80 molecules (details are available in Supplementary Materials Figure S2 and Table S1), with prominent compounds including esters (butanoic acid), alcohols (hex-(3Z)-enol, hexanol), aldehydes (nonanal), monoterpenes (β-pinene, 1,8-cineole, γ-terpinene), acyclic monoterpenes (myrcene, allo-ocimene), and oxygenated monoterpenes (linalool, camphor) (Table 6). Some of these compounds, such as β-pinene, 1,8-cineole, and linalool, were also found in fresh basil pesto sauce by the authors of [24].

The compounds identified in the pesto sauce formulations originate from the ingredients used in their preparation. For instance, compounds like 1,8-cineole, beta-pinene, linalool, (E)-2-hexenal, and camphor are derived from basil [25,28]. Moreover, hexanal and nonanal are present both in basil and extra virgin olive oil. In baru almonds, the presence of hexanal has also been reported [20]. In the case of moringa, several volatile compounds previously reported in its leaves, such as hexanal, trans-2-hexenal, hexanol, and benzaldehyde [55], were also identified in the corresponding pesto formulations, indicating their likely transfer from the raw material to the final product. It is noteworthy that moringa has a low content of volatile compounds [56], which is a positive factor for the development of pesto sauce with the addition of moringa leaves, as it minimizes alterations in the product’s aroma. In contrast, although baru almonds contain volatile compounds, particularly hexanal associated with nutty and peanut-like aromas, the low concentrations observed suggest that these compounds will not be perceptible in the formulation.

In the B/CN formulation 1,8-cineole emerged as the most abundant compound, constituting 21.1% of the relative peak area (RPA), closely followed by linalool (RPA = 16.4%) and camphor (RPA = 11.1%). The distinctive aroma of pesto sauce, primarily attributed to basil, is primarily shaped by these key volatiles [57]. Interestingly, the prevalence of 1,8-cineole remained consistent across other pesto sauce formulations as well, with RPAs of 23.7%, 22.8%, and 22.8% for B/BA, BM/CN, and BM/BA, respectively. On the other hand, there was a reduction in the content of linalool, especially in B/BA, which shows an RPA of 6.2%. Regarding camphor, the substitution of 50% of basil by moringa leaves slightly reduced its content (RPA of 8.8% and 9.4% for BM/CN and BM/BA, respectively). These results suggest that, in general, the inclusion of moringa leaves and baru almonds did not alter the principal aromatic compounds of the pesto sauces.

The presence of certain aldehydes and alcohols can be indicative of the extent of oxidative deterioration of a food sample as several volatile compounds resulting from the oxidation of fatty acids belong to the aldehydes and alcohols categories [58]. High levels of these volatile compounds, stemming from oxidation, may contribute to the development of “rancid” flavor and aroma in the food [59,60]. However, it is noteworthy that the absence or low percentage of RPA for these compounds was observed, with hexanal identified in the B/CN (2.0%) and BM/BA (4.6%), and nonanal in the B/BA formulation (0.2%). These results are favorable for the acceptability of pesto sauce, as a rancid flavor is commonly associated with food rejection.

### 3.5. Microbiological Analysis

The microbiological analysis was conducted before the sensory evaluation to ensure product safety, following the requirements established by Brazilian legislation. There were no differences among the pesto samples and the results for all samples were within satisfactory limits [61]. The counts of enterobacteria for all samples were lower than 1.0 × 10^1^ CFU/g Regarding Salmonella spp., all samples tested negative (absent in 25 g). As for filamentous fungi and yeast, the counts were 2.0 × 10^1^ CFU/g, 1.0 × 10^1^ CFU/g, 2.5 × 10^1^ CFU/g, 2.0 × 10^1^ CFU/g, and 2.5 × 10^1^ CFU/g for B/CN, B/BA, BM/CN, and BM/BA, respectively.

### 3.6. Sensory Evaluation

Sensory analysis plays an important role in assessing the impact of ingredient substitutions on products that are well received by consumers [62]. This enables an exploration of how the inclusion of UFPs influences the acceptance and perception of the product by consumers. The findings from the sensory analysis, particularly regarding acceptance tests and purchase intention of the pesto sauce formulations, are presented in Table 7.

The average hedonic scores provided by the consumer for the sensory attributes such as aroma, texture, flavor, and overall impression did not exhibit significant differences across all pesto sauces (*p* > 0.05). This indicates that the partial and/or total replacement of the traditional ingredients of the pesto sauce (basil leaves and cashew nuts) with moringa leaves and/or baru almonds did not alter the sensorial acceptability of the product by consumers, with average scores close to the hedonic score 7 (indicating “I liked it moderately”). This demonstrates that, regardless of the type of ingredient used, the set of compounds present in each formulation contributed to the development of the typical aroma, texture, and flavor characteristics of pesto sauce. This outcome can be partially attributed to the presence of (E)-2-hexenal, 1,8-cineole, linalool, and camphor, which were identified in all samples and contributed especially to the aromatic profile of the sauces. Furthermore, even the BM/BA formulation, which had a higher content of condensed tannins, compounds generally associated with increased astringency, was equally accepted compared to the others. Condensed tannins, especially those with a higher degree of polymerization, possess ortho di-hydroxy groups that favor the formation of hydrogen bonds with proteins, such as those present in saliva. This interaction can result in the formation of tannin–protein aggregates that precipitate, a process related to the perception of astringency [63]. However, this possible effect did not compromise the sensory acceptance of the formulation. Basil’s distinctive sensory characteristics, including a pleasant aroma and flavor widely appreciated in pesto sauce [64], likely contributed to the sustained acceptability, even with a 50% reduction in BM/CN and BM/BA formulations. Additionally, toasted baru almonds are recognized for their mild flavor [65], similar to peanuts, which could have positively influenced their acceptance. Pesto sauce thus represents a suitable food matrix for incorporating ingredients with sensory limitations, such as moringa, since its complex composition can help mask undesirable attributes like astringency.

The overall acceptability of pesto sauces produced with alternative ingredients, as well as the same intensity in the degree of acceptance compared to the traditional one (“I liked it moderately”; *p* ≥ 0.05), suggests that moringa leaves and baru almonds are viable ingredients for pesto sauce. Their inclusion expands the use of regional and underutilized ingredients, supporting both the valorization of non-conventional food plants (PANCs) and the development of nutritionally enhanced, sensorially accepted formulations with regional identity.

Concerning the attribute “appearance”, the B/CN formulation, which represents the traditional Brazilian pesto, exhibited lower acceptability than B/BA and BM/BA formulations (*p* < 0.05). This finding aligns with results from colorimetry analysis, where a significant difference in hue among the pesto sauce samples was revealed. As discussed in Section 3.2, the B/CN sample displayed a lesser tendency toward green compared to the other samples, potentially contributing to its lower acceptability. The reduction in green coloration may be associated with chlorophyll degradation, which can occur due to leaf aging or oxidative processes during processing and storage. This discoloration is recognized as an important indicator of quality in plant-based products. Moreover, in such products the pigment content, particularly chlorophyll, is directly linked to consumer preference [66]. The results emphasize the importance of appearance in influencing the acceptance of food by consumers.

In terms of purchase intention, no significant differences were observed among pesto sauces (*p* ≥ 0.05). Consumers assigned the terms “Probably would buy” and “Might buy” across all formulations. Consequently, the replacement of cashew nuts with baru almonds and the substitution of 50% of basil leaves with moringa leaves did not impact the purchase intention for the different pesto sauces. These findings emphasize the robustness of consumer acceptance and reinforce the feasibility of incorporating UFPs into traditionally well-accepted foods, thereby adding value to these products.

**Figure 1:**
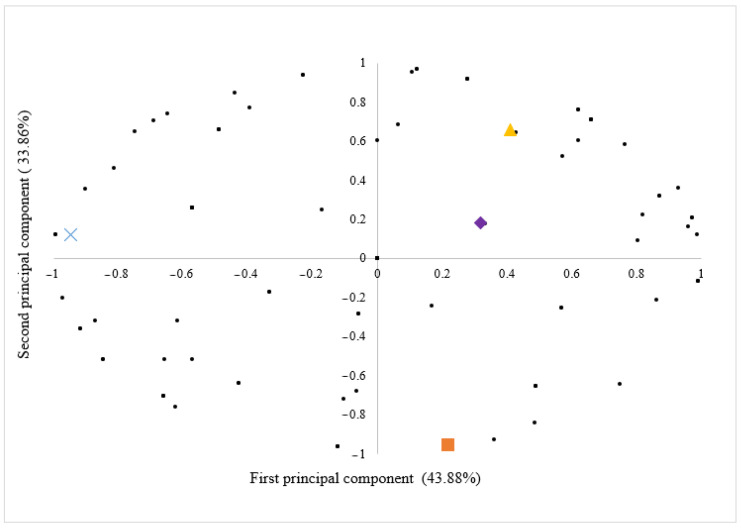
Preference map of the overall acceptability of the four pesto sauce formulations. (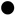) Consumers; (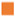) basil/cashew nut; (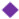) basil/baru almond; (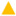) basil/moringa/baru almond; (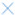) basil/moringa/cashew nut.

To gain a comprehensive understanding of individual consumer acceptability for the examined samples, we employed a preference map derived from the principal component analysis (PCA) based on the results of the overall impression acceptance test (Figure 1). This approach enables a more detailed analysis of consumer preferences, uncovering nuanced factors that influence the overall acceptability of the samples.

The first principal component explained 43.88% of the overall variability in acceptance across the formulations, with the second component contributing an additional 33.86%. Cumulatively, the two components explained 77.74% of the variance observed in the acceptability of pesto sauce formulations. Consequently, these two components were considered representative of the sample dispersion as they encompass the majority of the data variation.

In Figure 1, consumers are represented as points on the scatter plot, and their proximity to the sample labels (B/CN; B/BA; BM/CN; BM/BA) reflects a higher level of acceptance regarding overall impression. This observation underscores the uniformity in the distribution of consumers, with a comparable number of individuals represented for each pesto sauce sample. It is noteworthy that the graph contains overlapping points, making the perception of homogeneity difficult. Moreover, 11% of consumers are situated at the zero point in the graph, indicating a uniform acceptance of the samples by these consumers. This suggests that these consumers did not discern differences among the pesto sauce samples in relation to overall acceptability.

**Figure 2:**
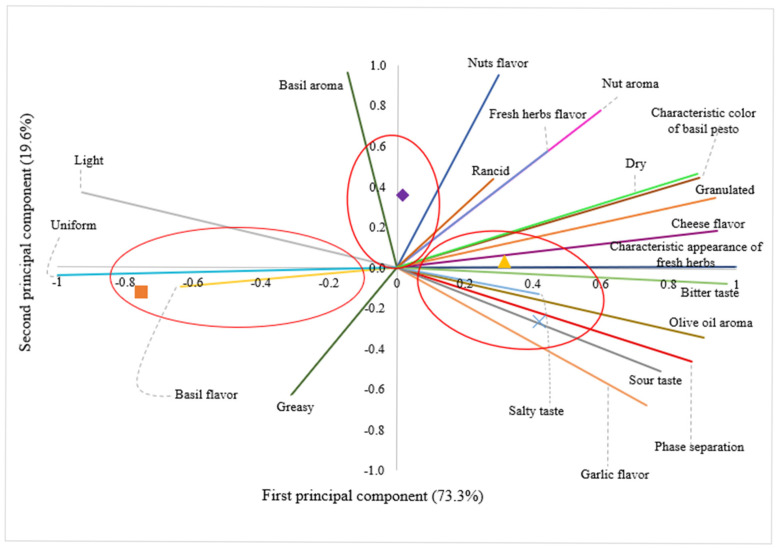
Descriptive sensory map obtained from pesto sauce formulations. (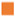) Basil/cashew nut; (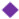) basil/baru almond; (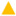) basil/moringa/baru almond; (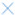) basil/moringa/cashew nut.

Descriptive sensory testing plays a crucial role in enhancing product quality, comparing formulations, identifying development issues, and aligning with consumer preferences. In this study, the “Rate-All-That-Apply” (RATA) test was employed, allowing consumers to identify and rate the intensity of sensory attributes in the sample. The correspondence analysis derived from the RATA results is shown in Figure 2. The various formulations of pesto sauces exhibited no significant differences in “basil aroma”, “nuts flavor”, or “greasy” attributes (*p* ≥ 0.05). Overall, the samples were grouped into the following three clusters: one group comprising the B/CN formulation, another containing the B/BA formulation, and a third group encompassing the BM/CN and BM/BA formulations. Notably, only the first principal component explained the variability of the samples regarding sensory attributes, accounting for 73.3% of the total variance.

The main attributes associated with the BM/CN and BM/BA samples were “characteristic appearance of fresh herbs”, “characteristic color of basil”, “phase separation”, “olive oil aroma”, “garlic flavor”, “salty taste”, “bitter taste”, “sour taste”, “cheese flavor”, “granulated”, and “dry”. Conversely, the main attributes associated with the B/CN sample were “light”, “uniform”, and “basil flavor”. As for B/BA, it was related to intermediate characteristics of the other two groups. Based on the sensory attributes associated with the samples, it can be inferred that although the BM/BA sample was described as having the typical appearance and color of fresh herbs and basil, and the B/BA sample was associated with intermediate characteristics of these attributes, both showed higher appearance acceptance levels compared to the B/CN group. In the case of the BM/CN group, the absence of differences in appearance acceptance, despite variations in the sensory attributes associated with the samples, indicates that a variety of sensory descriptors contribute to the acceptance of pesto sauce by consumers. Another factor is that despite the BM/CN and BM/BA samples showing a reduction in basil, this change did not affect consumer acceptability. The findings suggest that incorporating alternative sources, such as moringa leaves and baru almonds, into pesto sauce is a viable option. Despite generating varying perceptions regarding specific descriptive attributes, the inclusion of these alternative ingredients did not lead to rejection by consumers. In fact, their acceptance was either equivalent or superior to that of the traditional sample (B/CN).

The development of foods from sustainable sources is a relevant strategy to meet new consumption trends and the nutritional demands of the population. The search for unconventional plant-based food sources holds great potential for expanding the food supply chain with a lower environmental impact [2]. The use of moringa leaves and baru almonds has significant implications for the development of food products, contributing to the availability of more sustainable foods with reduced environmental impact, in line with the preferences of environmentally conscious consumers.

The baru almond is considered to be the most economically important and edible part of the baru fruit. Its use is promising as its nutritional and sensory characteristics make it highly applicable in various food products [67]. The almond can be used in different forms as its oil can be extracted and incorporated into formulations, the defatted flour can be used in baked goods, and the whole almond can be included in cereal bars, allowing its incorporation into a wide range of food matrices. The leaves of *Moringa oleifera* can be used both fresh and dried, with various applications in food products such as soups, pasta, and other preparations. Due to their high nutritional quality and contribution to sustainability, incorporating these leaves into food products represents a promising market strategy, with the potential to attract consumers interested in functional and sustainable foods. In general, the use of moringa leaves and baru almonds contributes to increasing food diversity and improving consumer access to nutritious and varied foods.

## 4. Conclusions

The combination of moringa leaves and baru almonds into pesto sauce enhanced the nutritional profile by increasing the protein content and reducing the fat level without compromising sensory acceptance or consumer purchase intent. The incorporation of these UFPs also influenced color attributes, enhancing the green hue; however, they did not significantly impact the concentration of total phenolic compounds and the antioxidant capacity in the pesto sauces.

The key volatile compounds responsible for the characteristic aroma of pesto, such as 1,8-cineole and linalool, were preserved across all formulations, indicating that the inclusion of moringa and baru did not detract from the product’s aromatic identity. Furthermore, the use of the descriptive sensory technique RATA revealed that the essential sensory attributes in pesto sauce, including a basil aroma and nutty flavor, remained consistent between the reformulated and traditional samples.

Overall, pesto sauce proved to be a viable food matrix for incorporating underutilized regional ingredients, offering a sustainable and consumer-accepted alternative for food product innovation.

## Figures and Tables

**Table 1:** Pesto sauce formulations.

Pesto Ingredients (g/100 g)	B/CN	B/BA	BM/CN	BM/BA
Basil leaves	22.0	22.0	11.0	11.0
Moringa leaves	-	-	11.0	11.0
Cashew nuts	11.0	-	11.0	-
Baru almonds	-	11.0	-	11
Extra virgin olive oil	54.0	54.0	54.0	54.0
Parmesan cheese	10.0	10.0	10.0	10.0
Galic	2.0	2.0	2.0	2.0
Salt	1.0	1.0	1.0	1.0

Note: B/CN, the traditional Brazilian version featuring basil and cashew nuts; B/BA, a formulation substituting cashew nuts with baru almonds; BM/CN, replacing 50% of the basil with moringa leaves, plus cashew nuts; BM/BA, replacing 50% of the basil with moringa leaves, plus baru almonds.

**Table 2:** Proximate composition of basil and moringa leaves, cashew nuts, and baru almonds.

	Component (% *w*/*w*)				
Pesto Ingredients	Moisture	Carbohydrate	Protein	Lipid	Ash
Basil leaves	88.4 ± 0.4 ^a^	6.3 ± 0.1 ^b^	3.4 ± 0.4 ^b^	0.5 ± 0.1 ^b^	1.4 ± 0.1 ^b^
Moringa leaves	70.0 ± 0.2 ^b^	12.9 ± 0.3 ^a^	10.3 ± 0.7 ^a^	3.2 ± 0.1 ^a^	2.6 ± 0.1 ^a^
Cashew nuts	3.1 ± 0.0 ^B^	25.3 ± 0.1 ^B^	20.3 ± 0.7 ^B^	49.1 ± 0.7 ^A^	2.2 ± 0.0 ^B^
Baru almonds	6.4 ± 0.3 ^A^	26.1 ± 0.3 ^A^	26.5 ± 2.3 ^A^	38.3 ± 0.0 ^B^	2.7± 0.0 ^A^

Different lowercase letters indicate statistical differences between leaves and different uppercase letters indicate differences between cashew nuts and almonds by Tukey’s test. Significance level *p* < 0.05. Coefficient of variation < 10.

**Table 3:** Proximate composition of pesto sauce formulations.

	Component (% *w*/*w*)				
Pesto Sauces	Moisture	Carbohydrate	Protein	Lipid	Ash
B/CN	22.7 ± 0.0 ^b^	6.9± 0.3 ^b^	7.9 ± 0.4 ^b^	60.1± 0.3 ^a^	2.4 ± 0.0 ^b^
B/BA	25.0 ± 0.5 ^a^	7.7 ± 0.6 ^ab^	8.7 ± 0.3 ^ab^	55.9 ± 0.6 ^b^	2.7 ± 0.1 ^a^
BM/CN	21.9 ± 0.6 ^b^	7.4 ± 0.3 ^ab^	8.0 ± 0.3 ^b^	60.2 ± 0.3 ^a^	2.5 ± 0.1 ^b^
BM/BA	22.6 ± 0.2 ^b^	8.7 ± 0.3 ^a^	9.0 ± 0.2 ^a^	56.9 ± 0.3 ^b^	2.8 ± 0.0 ^a^

Different letters indicate statistical differences within the column by Tukey’s test. Significance level *p* < 0.05. Coefficient of variation < 10. B/CN, the traditional Brazilian version featuring basil and cashew nuts; B/BA, a formulation substituting cashew nuts with baru almonds; BM/CN, replacing 50% of the basil with moringa leaves, plus cashew nuts; BM/BA, replacing 50% of the basil with moringa leaves, plus baru almonds.

**Table 4:** Mean values ± standard deviation for the chromatic coordinates L* (luminosity), a* (chromaticity), and b* (chromaticity), as well as for the values of saturation index (C*), hue angle (h°), and color variation parameter (ΔE).

Pesto Sauces	L*	a*	b*	h°	C*	ΔE
B/CN	30.2 ± 0.7 ^a^	0.1 ± 0.1 ^a^	22.5 ± 0.5 ^a^	89.5 ± 0,2 ^d^	22.5 ± 0.5 ^a^	-
B/BA	28.7 ± 0.4 ^ab^	−1.4 ± 0.1 ^b^	22.8 ± 0.7 ^a^	93.5 ± 0.2 ^c^	22.9 ± 0.7 ^a^	2.4 ± 0.1 ^b^
BM/CN	27.2 ± 1.2 ^ab^	−3.0 ± 0.6 ^c^	22.3 ± 3.1 ^a^	97.7 ± 0.7 ^b^	22.5 ± 3.1 ^a^	5.4 ± 0.1 ^a^
BM/BA	26.3 ± 1.3 ^b^	−4.1 ± 0.5 ^c^	19.8 ± 0.5 ^a^	101.6 ± 1.6 ^a^	20.2 ± 0.4 ^a^	6.4 ± 0.5 ^a^

Different letters indicate statistical differences within the column by Tukey’s test. Significance level *p* < 0.05. B/CN, the traditional Brazilian version featuring basil and cashew nuts; B/BA, a formulation substituting cashew nuts with baru almonds; BM/CN, replacing 50% of the basil with moringa leaves, plus cashew nuts; BM/BA, replacing 50% of the basil with moringa leaves, plus baru almonds.

**Table 5:** Mean total phenolic compounds (TPCs) (expressed in mg of gallic acid equivalent (GAE)/100 g sample), condensed tannins (expressed in mg of catechin equivalents per gram of sample (CE)/100 g sample), and antioxidant capacity determined by the ABTS and FRAP methods for pesto sauce samples.

Pesto Sauces	TPCs (mg GAE/100 g)	Condensed Tannins (mg CE/100 g)	ABTS (mg Trolox Equivalent/100 g)	FRAP (mg Trolox Equivalent/100 g)
B/CN	151.8 ± 4.5 ^a^	50.7 ± 2.8 ^c^	304.9 ± 14.1 ^a^	128.6 ± 9.4 ^a^
B/BA	93.1 ± 2.8 ^b^	43.8 ± 0.8 ^c^	167.7 ± 13.9 ^b^	75.6 ± 8.4 ^b^
BM/CN	150.8 ± 11.6 ^a^	86.1 ± 6.0 ^b^	289.4 ± 13.2 ^a^	112.7 ± 3.9 ^a^
BM/BA	162.7 ± 7.4 ^a^	113.3 ± 1.4 ^a^	287.4 ± 5.2 ^a^	133.3 ± 8.4 ^a^

Different letters indicate statistical difference within the column. Significance level *p* < 0.05, by Tukey’s test. Coefficient of variation < 10. B/CN, the traditional Brazilian version featuring basil and cashew nuts; B/BA, a formulation substituting cashew nuts with baru almonds; BM/CN, replacing 50% of the basil with moringa leaves, plus cashew nuts; BM/BA, replacing 50% of the basil with moringa leaves, plus baru almonds.

**Table 6:** Main volatile compounds, retention index, and percentage of peak area identified in pesto sauce.

Component	Retention Index				Relative Peak Area (%)			
	B/CN	B/BA	BM/CN	BM/BA	B/CN	B/BA	BM/CN	BM/BA
Hex-(2E)-enal	854.0	854.0	854.0	855.0	4.4	3.6	8.8	8.6
1,8-cineole	1037.0	1036.0	1035.0	1036.0	21.1	23.7	22.8	22.8
Linalool	1104.0	1101.0	1101.0	1102.0	16.4	6.2	10.0	11.6
Camphor	115.7	1151.0	1151.0	1151.0	11.5	14.1	8.8	9.4

B/CN, the traditional Brazilian version featuring basil and cashew nuts; B/BA, a formulation substituting cashew nuts with baru almonds; BM/CN, replacing 50% of the basil with moringa leaves, plus cashew nuts; BM/BA, replacing 50% of the basil with moringa leaves, plus baru almonds.

**Table 7:** Mean hedonic scores and purchase intent of pesto sauce formulations.

Pesto Sauces	Appearance	Aroma	Texture	Flavor	Global Impression	Purchase Intent
B/CN	6.5 ± 2 ^b^	6.9 ± 1.5 ^a^	7.1 ± 1.6 ^a^	7.1 ± 1.7 ^a^	6.9 ± 1.4 ^a^	3.3 ± 1.1 ^a^
B/BA	7.0 ± 1.8 ^a^	7.1 ± 1.6 ^a^	7.2 ± 1.6 ^a^	7.2 ± 1.6 ^a^	7.2 ± 1.3 ^a^	3.5 ± 1.1 ^a^
BM/CN	6.9 ± 1.8 ^ab^	7.1 ± 1.6 ^a^	7.0 ± 1.5 ^a^	7.2 ± 1.6 ^a^	7.1 ± 1.5 ^a^	3.5 ± 1.1 ^a^
BM/BA	7.1 ± 1.7 ^a^	7.0 ± 1.7 ^a^	6.9 ± 1.7 ^a^	7.1 ± 1.6 ^a^	7.1 ± 1.4 ^a^	3.5 ± 1.1 ^a^

Means with different lowercase letters in the column indicate a significant difference by Tukey’s test (*p* < 0.05). B/CN, the traditional Brazilian version featuring basil and cashew nuts; B/BA, a formulation substituting cashew nuts with baru almonds; BM/CN, replacing 50% of the basil with moringa leaves, plus cashew nuts; BM/BA, replacing 50% of the basil with moringa leaves, plus baru almonds.

## Data Availability

The original contributions presented in this study are included in the article. Further inquiries can be directed to the corresponding author.

## Supplementary Materials

The following supporting information can be downloaded at: https://www.mdpi.com/article/10.3390/foods14132377/s1, Figure S1: Model of the evaluation forms used in the acceptability, purchase intention and sensory characterization analyzes of pesto sauce formulations; Figure S2: Chromatograms of pesto sauce samples and alkane standards; Table S1: Volatile compounds, retention index, and percentage of peak area identified in pesto sauce.
