# Supplementary material for: Development of Pesto Sauce with Moringa Leaves and Baru Almonds: A Strategy to Incorporate Underutilized Ingredients with Nutritional and Sensory Viability
Source: Foods. 2025 Jul 4;14(13):2377. doi: 10.3390/foods14132377 (PMC12248746; doi:10.3390/foods14132377)
Supplement: Supplementary file 1 [file foods-14-02377-s001.zip › foods-3725180-supplementary.pdf]

Development of pesto sauce with moringa leaves and baru almonds: a strategy to incorporate underutilized ingredients with nutritional and sensory viability

SUPPLEMENTARY MATERIAL

RATA

Name:______________ Gender: F ( ) M ( ) Year of birth: ______Date:_________

Please evaluate the appearance and aroma of the sample, and then taste it. Identify (mark with an X) your opinion regarding the intensity of the attributes according to the scale below:

Sample:___________

	**Attributes**	**Not applicate**	**Low**	**Medium**	**High**
Appearance	Characteristic color of basil pesto	( )	( )	( )	( )
	Phase separation	( )	( )	( )	( )
	Characteristic appearance of fresh herbs	( )	( )	( )	( )
Aroma	Olive oil aroma	( )	( )	( )	( )
	Nut aroma	( )	( )	( )	( )
	Basil aroma	( )	( )	( )	( )
Flavor	Garlic flavor	( )	( )	( )	( )
	Sour taste	( )	( )	( )	( )
	Bitter taste	( )	( )	( )	( )
	Nut flavor	( )	( )	( )	( )
	Fresh herbs flavor	( )	( )	( )	( )
	Rancid	( )	( )	( )	( )
	Light	( )	( )	( )	( )
	Basil flavor	( )	( )	( )	( )
	Cheese flavor	( )	( )	( )	( )
	Salty taste	( )	( )	( )	( )

	Greasy	( )	( )	( )	( )
Texture	Uniform	( )	( )	( )	( )
	Dry	( )	( )	( )	( )
	Granular	( )	( )	( )	( )

ACCEPTANCE

Please rate using the scale below how much you liked or disliked the product in relation to its attributes:

9 - I Liked extremely
8 - I Liked very much
7 - I Liked moderately
6 - I Liked slightly
5 - Indifferent
4 - Dislike slightly
3 - Dislike moderately
2 - Dislike very much
1 - Dislike extremely

Appearance: _________
Aroma: _____________
Texture: ____________
Flavor: _____________
Global impression:___________

Purchase intent

( ) Certainly would buy
( ) Probably would buy
( ) Might buy
( ) Probably would not buy
( ) Certainly would not buy

Comments:

Figure S1. Model of the evaluation forms used in the acceptability, purchase intention and sensory characterization analyzes of pesto sauce formulations.

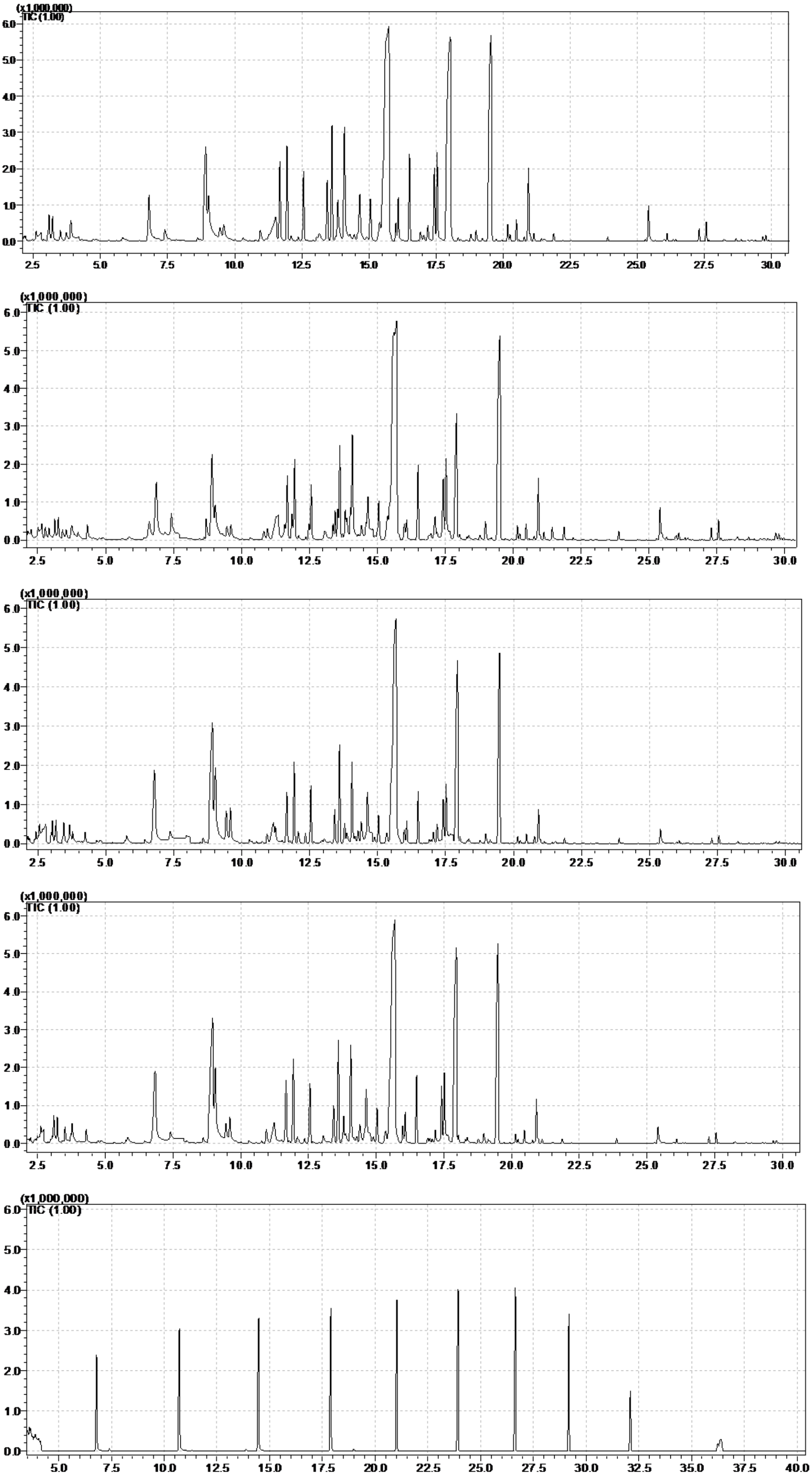

A

B

C

D

E

Figure S2. Chromatograms of pesto sauce samples and alkane standards. (A) basil/cashew nut formulation; (B) basil/baru almond formulation; (C)

basil/moringa/cashew nut formulation; (D) basil/moringa/baru almond formulation; (E) alkane standards.

Table S1. Volatile compounds identified in the pesto formulations and their distribution among the samples.

**Compound**	**Retention Index**	**B/CN**	**B/BA**	**BM/CN**	**BM/BA**
Hexanal	800.4-801.2	✓	–	–	✓
Ethyl butyrate	802.0	–	✓	–	–
Trans-2-hexenal	846.0-847.0	✓	–	–	✓
Hex-(3Z)-enol	857.0-858.0	–	✓	✓	✓
Hex-(3Z)-enyl formate	857.0	✓	–	–	–
Hex-(2E)-enol	868.0	✓	–	✓	✓
Trans-2-Hexen-1-ol	868.0	–	✓	–	–
Hexanol	871.0-872.0	✓	✓	✓	✓
Dimethyldiethoxysilane	903.0	–	✓	–	–
Methional	906.0	✓	✓	✓	✓
Benzenemethanol, ar,ar,.α.-trimethyl	912.0-922.0	✓	✓	✓	–
2-allyl-6-methylpyrazine	914.0	–	–	✓	–
Tricyclene	921.0	–	–	–	✓
Origanene	925.0-926.0	✓	✓	✓	✓
α-pinene	932.0-933.0	✓	✓	✓	✓
Cyclopropane, 1,1-dimethyl-2-(2-methyl-2-propenyl)	937.0	✓	✓	✓	✓
Camphene	949.0	✓	✓	✓	✓
(S*,S*)-2-Hydroxy(4-methoxy-2-trimethylsilylphenyl)methyl-1-cycloheptanone	960.0	–	✓	–	–
Benzaldehyde	962.0	✓	–	✓	✓
Methanone, (dimethylphenyl)phenyl	963.0	–	✓	–	–
2,3-dihydro-6-oxo-7-(trimethylsilyl)-6H-5-oxaindene	965.0	✓	–	–	–
Sabinene	972.0-973.0	✓	✓	✓	✓
β-Pinene	977.0	✓	✓	✓	✓
Vinyl amyl carbinol	982.0-983.0	✓	✓	✓	✓
Myrcene	989.0-990.0	✓	✓	✓	✓

1,5-Heptadiene, 2-ethyl-6-methyl	995.0-996.0	✓	–	✓	✓
Ethyl hexanoate	998.0	–	–	✓	✓
Decane	1000.0	✓	–		
3-Hexen-1-ol, acetate, (Z)-	1005.0	–	–	✓	✓
spiro[5.2]octan-1,1-Dicarbonitrile, 2-methyl-	1005.0-1006.0	✓	✓	–	–
Hexyl acetate	1012.0-1013.0	✓	–	✓	✓
Terpinene <α->	1016.0-1017.0	✓	✓	✓	✓
Cymene <p->	1025.0-1027.0	✓	–	✓	✓
Phenylacetaldehyde	1044.0-1045.0	✓	✓	✓	✓
Ocimene <(E)-, beta->	1047.0	✓	✓	✓	✓
.Γ.-terpinene	1059.0-1060.0	✓	✓	✓	✓
Trans Sabinene hydrate	1071.0-1072.0	✓	–	✓	–
N-octanol	1076.0	✓	–	–	–
Tetrasulfide <diallyl->	1080.0	–	–	✓	✓
Terpinolene	1086.0-1087.0	✓	✓	✓	✓
Fenchone	1089.0-1090.0	✓	✓	✓	✓
Nonanal	1004.0		✓	–	–
Nona-1,3,7-triene <4,8-dimethyl-, (E)->	1113.0	✓	✓	–	–
Phenethyl alcohol	1115.0-1117.0	–	✓	–	✓
Benzeneethanol	1115.0	–	–	✓	–
Campholenic aldehyde <α->	1128.0-1129.0	✓	✓	✓	✓
Cyclopentasiloxane, decamethyl	1135.0	✓	✓	✓	✓
Allyl methyl trisulfide	1140.0	–	–	✓	✓
Allo-ocimene	1143.0	✓	–	–	–
Terpineol <δ->	1172.0-1173.0	✓	✓	✓	✓
Endo-Borneol	1175.0	–	✓	✓	✓
Borneol	1175.0	✓			
Terpinen-4-ol	1182.0-1183.0	✓	✓	✓	✓
3-vinyl-[4H]-1,2-dithiin	1192.0	✓	✓	✓	✓
(-)-.A.-terpineol	1197.0	✓	–	–	–

Terpineol <α->	1196.0-1197.0	–	✓	✓	✓
1-Tridecene	1203.0	–	–	✓	✓
3-Tetradecene, (Z)	1204.0	✓	–	–	–
1-Pentadecene	1204.0	–	✓	–	–
2H-1,4-Benzodiazepin-2-one, 7-chloro-1,3-dihydro-5-phenyl-1-(trimethylsilyl)-	1229.0	✓	–	–	–
7-chloro-1,3-dihydro-5-phenyl-2H-1,4-dibenzodiazepin-2-thione	1229.0	–	✓	✓	✓
5,6-exo-Epoxy-N-phenyl-1-trimethylsilylmethylbicyclo-[3.2.1]heptane-2,3-endo-dicarboximide	1241	–	✓	–	–
Eugenol	1354.0-1355.0	✓	✓	✓	✓
Copaene <α->	1380.0	✓	✓	✓	✓
A.-bourbonene	1388.0		✓		
Caryophyllene <(E)->	1425.0-1426.0	✓		✓	✓
Bergamotene <α-, cis->	1436.0	✓	✓	✓	✓
.α.-amorphene	1479.0-1480.0	✓	✓	–	–
Muurolene <γ->	1516.0	–	✓	–	–
Cadinene <γ->	1516.0-1517.0	✓	–	–	✓
Cadinene <δ->	1520.0	✓	✓	–	✓
